# What drives horse success at following human-given cues? An investigation of handler familiarity and living conditions

**DOI:** 10.1007/s10071-023-01775-0

**Published:** 2023-04-19

**Authors:** Océane Liehrmann, Camille Cosnard, Veera Riihonen, Alisa Viitanen, Emmi Alander, Plotine Jardat, Sonja E. Koski, Virpi Lummaa, Léa Lansade

**Affiliations:** 1 Department of Biology, University of Turku, Vesilinnantie 5, Natura Building, 20500 Turku, Finland; 2 CNRS, IFCE, INRAE, Université de Tours, PRC, 37380 Nouzilly, France; 3 Organismal and Evolutionary Biology Research Programme, University of Helsinki, 00014 Helsinki, Finland

**Keywords:** Social cognition, Human–animal relationship, Pointing, Housing condition, *Equus caballus*, Welfare, Herd size

## Abstract

Cues such as the human pointing gesture, gaze or proximity to an object are widely used in behavioural studies to evaluate animals’ abilities to follow human-given cues. Many domestic mammals, such as horses, can follow human cues; however, factors influencing their responses are still unclear. We assessed the performance of 57 horses at a two-way choice task testing their ability to follow cues of either a familiar (*N* = 28) or an unfamiliar informant (*N* = 29). We investigated the effects of the length of the relationship between the horse and a familiar person (main caregiver), their social environment (living alone, in dyads, or in groups) and their physical environment (living in stalls/paddocks, alternating between paddocks and pastures, or living full time in pastures). We also controlled for the effects of horses’ age and sex. Our results showed that horses’ success rate at the task was not affected by the familiarity of the informant and did not improve with the relationship length with the familiar informant but did increase with the age of the horses. Horses living in groups had better success than the ones kept either in dyads or alone. Finally, horses housed in small paddocks had lower success than those living on pasture. These results indicate that with age, horses get better at following human-given indications regardless of who the human informant is and that an appropriate living and social environment could contribute to the development of socio-cognitive skills towards humans. Therefore, such aspects should be considered in studies evaluating animal behaviour.

## Introduction

Humans have been working with animals for thousands of years. Communication between humans and animals in such situations relies on the animal’s ability to understand human-given cues. Gestural cues are used by humans for referential communication to share intentions and the manual pointing is one of the first communicative gestures to appear in children (Leavens and Hopkins 1999). Because of their central role in human communication, gestural cues are now widely used in animal cognitive experiments to investigate how animals understand and interpret cues from humans. In such tests, a person points towards an object where food is hidden, and the animal subject has to use the human-given cues to make the correct choice between this object and at least one other identical object to receive a reward.

Many domestic mammals, such as cats, dogs, ferrets, pigs and horses, have been found to be able to use human referential communication (Jardat and Lansade 2021; Krause et al. 2018), but most studies have focussed on dogs and horses. Whereas dogs are clearly the most skilful, the results from horses are more mixed. One study showed that horses were better at responding to a pointing signal if the hand was close to the target and if it was held there until the animal made the choice (Maros et al. 2008). Proops et al. (2010) revealed that horses were able to use pointing gestures and object placement cues but could not rely on just body orientation or gaze. Such performance variation between studies and between individuals may not only come from the quality or the type of cues used by humans to communicate with horses but may be the results of external factors such as the informant identity or the horse environment.

First, informant identity may affect horses’ ability or willingness to respond to human-given cues; in dogs, familiarity has been shown to enhance their ability to follow human-given cues (Cook et al. 2014; Cunningham and Ramos 2014; Scandurra et al. 2017). In horses, Ringhofer et al. (2021) observed that they would choose to follow the pointing of someone they witnessed being informed of where the food was hidden. In 2011, Krueger et al. observed that horses walked more often towards the focus of attention of a familiar experimenter compared to that of an unfamiliar experimenter in a three-way object-choice task. They suggest that socialisation with humans may improve horse abilities to read human-given cues. To our knowledge, this is the first and only report on the effect of the informant’s familiarity during choice tasks in horses. Previous studies have shown that horses are very good at recognising individual humans based on holistic information and not just simple cues such as hair colour (Lansade et al. 2020), which suggests that the informant’s identity and familiarity can be of great importance to the experimental design. However, familiarity is quite a general term for defining specified human-horse relationships, as these relationships can vary from occasional interactions to a long-term bond (Hausberger et al. 2008). One way to be more specific is to consider the relationship length between the familiar person and the animal. In working Asian elephants, the relationship length affected the elephants’ response in test situations; the elephants agreed more often to step on a novel surface and responded faster when they were called by a familiar handler whom they had known for a longer time (Crawley et al. 2021; Liehrmann et al. 2021). In horses, recent findings from Liehrmann et al. (2022) show that older horses agreed more often to walk on a novel surface when led by someone familiar than someone unfamiliar, and in general horses with a longer relationship with their owner were less scared of novelty than horses having a shorter relationship with their handler. Thus, one of the aims of this study was to investigate how the familiarity and the relationship length between a horse and its main caregiver affected the horse’s response in a pointing task.

Second, the housing system may play an important role affecting animals’ ability or willingness to respond to human-given cues. Numerous studies have highlighted the positive effects of environmental enrichment and voluntary physical exercise on neurogenesis, learning and memory in animals (Bekinschtein et al. 2011; van Praag et al. 2000). Therefore, when exploring the socio-cognitive skills of social species, the social and physical environment in which they are living is also important to consider. Many studies have observed the negative impacts of social deprivation on the cognitive abilities of social species (Ashton et al. 2018a; Lambert and Guillette 2021), but the effect of the social and physical environments on cognitive performance has rarely been studied in long-lived mammals. Moreover, to our knowledge, the effect of sociality with conspecifics has not been investigated in association with socio-cognitive skills towards humans. Horses naturally live in complex social groups (Klingel 1982; Ransom and Kaczensky 2016) and free-ranging feral horses can move distances averaging from 9 to 16 km daily and cover areas up to 40 km^2^ in one summer (Hampson et al. 2010; Henning et al. 2018). In contrast, domestic horses are kept in enclosures varying in size and in the number of conspecifics, or in individual stalls or paddocks. In bigger pastures, horses are more active compared to in small paddocks (Maisonpierre et al. 2019). They are free to move according to their needs – e.g. to look for shade or a shelter against wind and rain – and have enough space to exercise at will. It has been shown that horses with access to pasture with conspecifics showed better learning performance (Lansade et al. 2014a, b) and had a better relationship with humans (fewer displays of aggression) (Ruet et al. 2020; Søndergaard and Ladewig 2004) than horses kept in individual stables. Thus, we wanted to explore whether the social environment of individuals may also affect horses’ socio-cognitive abilities towards humans.

This study aims to investigate extrinsic factors that could impact a horse’s success at following human-given cues in a two-way object-choice task according to the familiarity of the human informant (main caretaker of the horse or an unfamiliar experimenter). We used a global cue—combining pointing gesture, gaze in the direction of the target object and proximity to the object- that mimics a signal close to what humans would naturally use to communicate with their animal. We compared the horses’ performance at following this cue according to the familiarity of the informant as well as their relationship length with the horse and the physical and social environment of the horse. (1) The main hypothesis is that horses perform better with a familiar informant than with an unfamiliar informant (Krueger et al. 2011). (2) When the given cues are performed by a familiar informant (main caretaker of the horse), we expect the horses’ success to improve with the length of their relationship (Liehrmann et al. 2021, 2022). We do not expect the relationship length with the main caretaker to affect the horses’ performances when tested with an unfamiliar informant. (3) When testing the effect of the social and physical environment of the horses, we hypothesise that horses living in groups or in dyads would show better success than horses living alone due to the benefit of sociality on cognitive abilities (Ashton et al. 2018a; Lambert and Guillette 2021). (4) We hypothesise that horses with access to bigger fields would show more success than horses living in smaller paddocks due to the benefit of a more enriched environment on cognitive abilities (Bekinschtein et al. 2011; van Praag et al. 2000). We do not expect the familiarity of the informant to interfere with the potential effects of the social and physical environment. In all analyses, we also controlled for the effect of the sex and age of the horses. (5) Finally, we expect a learning effect through the trials, and we hypothesise that horses tested with a familiar informant will learn to follow the cues faster than horses tested with an unfamiliar informant.

## Materials and methods

### Study population and collected information

We used advertisements on social media to recruit 52 volunteers and their leisure horse(s) for participation in the study. The condition to participate was that the horse had received enough training to walk safely on a leash in a familiar environment. The horses were located in 26 different places (private homes or private stables) in Southern Finland. A total of 72 horses participated in the study, of which 57 individuals passed a training phase (see below) and could be tested (29 females and 28 males–27 geldings, 1 stallion). Data from these 57 individuals were used for the analyses. The horses ranged in age from 2 to 26 years (mean ± SD = 12.21 ± 5.56) and were of mixed breeds (see Liehrmann et al. 2022 in the section Availability of data and materials for details). The familiarity of the experimenters, the length of the relationship between the horse and the familiar person, and the categories defining the social and physical environment of the horses are presented in Table 1, and the sample sizes according to the familiarity of the experimenter performing the pointing test are presented in Table 2.

**Table 1:**
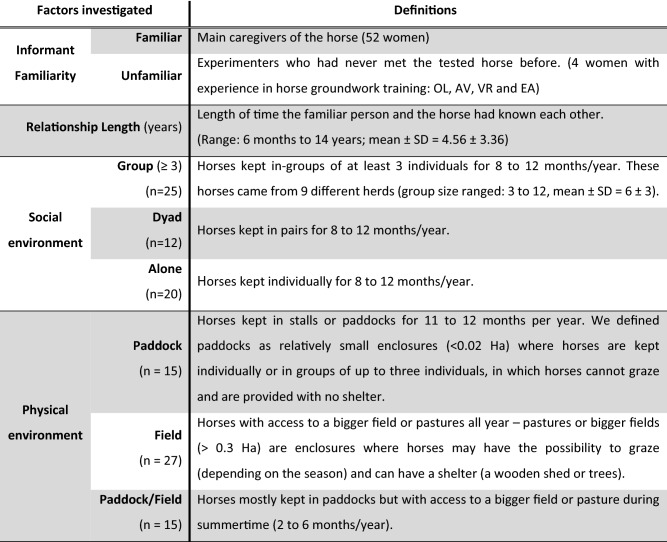
Definitions of the main investigated factors

**Table 2:**
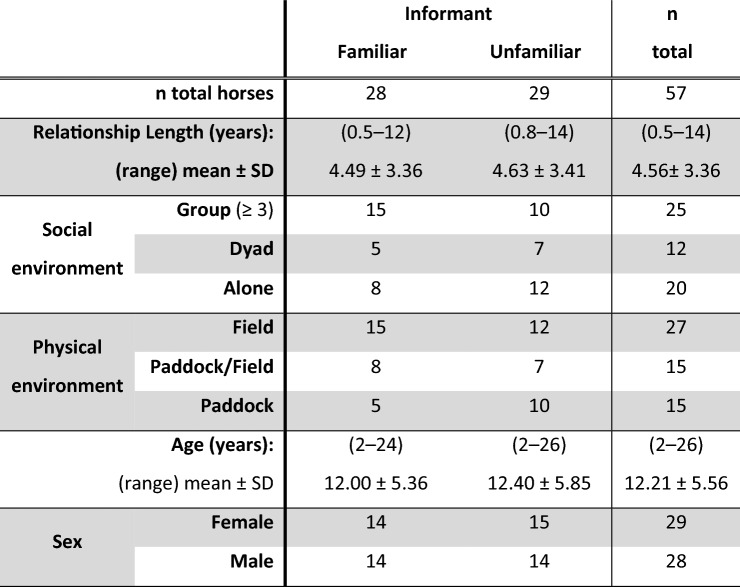
Sample size according to the familiarity of the informant and five other variables used in the analyses

### Experimental design

The experiments were carried out between March and April 2021 in stables in Southern Finland in which the horses lived. The tests were performed in a place familiar to the horses (riding arena or empty paddock).

#### The training phase

**Fig. 1:**
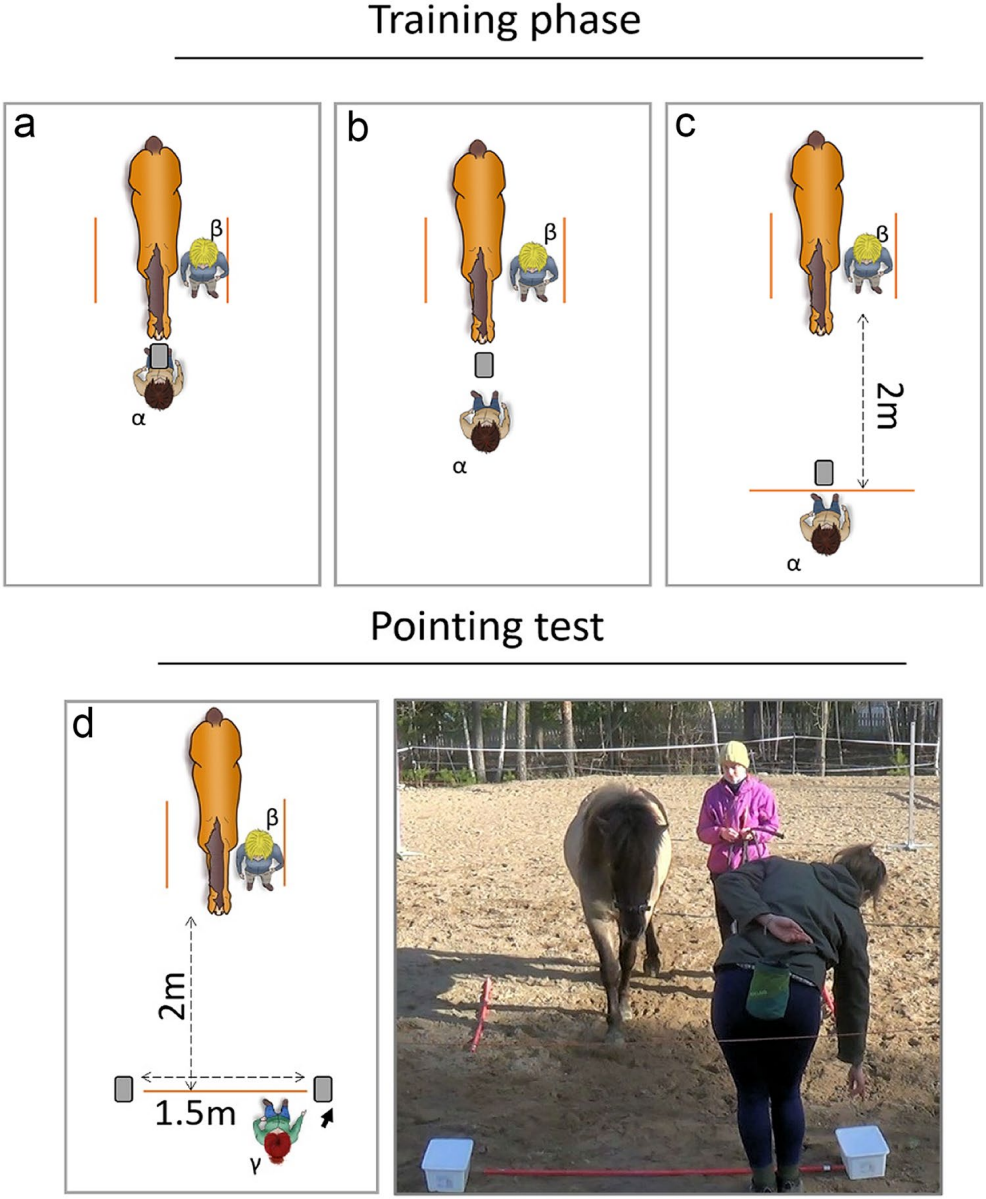
Training phase and Pointing test layout. **a** Training phase “a”, **b** training phase “b”, **c** training phase “c” also used as a motivational trial during the pointing test, **d** pointing test with an example of a pointing trial. Picture presenting the real condition of d. “α”: Unfamiliar experimenter performing the training phase. “β”: unfamiliar research assistant. “γ”: informant (Familiar or Unfamiliar person but different from the trainer)

The goal of the training phase was to inform the horse that it could get food from a bucket and to assess its motivation to move towards the buckets on its own. First, in the training phase *a* (Fig. 1a), the horse was led by one of the four unfamiliar experimenters towards the starting point and stopped. A bucket with a small piece of carrot (2 cm) inside was then presented to the horse, held by another unfamiliar experimenter, and the horse was free to eat the carrot. Then, a lid was put on the bucket, and the horse had to touch it with the muzzle for the bucket to be opened by the trainer. Training phase *a,* with the lid on, was repeated three times in a row. Second, during training phase *b* (Fig. 1b), the bucket was no longer held by the trainer, but instead placed on the floor 50 cm in front of the horse. The horse was led to the starting point and released so that it could go to touch the bucket for the trainer to open the lid. Training phase b was repeated three times. Training phase *c* (Fig. 1c) was similar to phase *b* but with the bucket two metres away from the starting point and the trainer standing behind the bucket. The horse was led to the starting point and released, and it then had to move towards the bucket and touch the lid with the muzzle for it to be opened by the trainer. Three to six trials of phase *c* were performed one after another. The horse needed to approach and touch the lid of the bucket three times in a row to be selected for the pointing test. If the horse did not satisfy the criterion within six trials, it was not selected for the test phase. Altogether, 15 horses failed to pass the training phase while 57 horses passed the training phase and are examining in this study. The 15 horses which dropped out of the study do not appear to be systematically associated with any of the parameters we are looking at in this study. Four of them were housed in groups, six in paddock and five alternated both. Similarly, six were kept alone, four in dyads and five in groups. Their age ranged from 2 to 24 years old and the relationship with the main caretaker from 4 to 15 years long, and they were composed of seven females and eight males. We observed that these 15 horses were not motivated by the food reward. To avoid a preliminary association of the person with a food reward, the training phase was always performed by an unfamiliar experimenter, and the pointing test was then performed by a different unfamiliar informant or a familiar informant.

#### The pointing test

Two buckets with a lid were placed 1.5 m apart on both sides of the informant experimenter. The approach and starting point were the same as in the training phase *c* (Fig. 1c, d). Both buckets had a piece of carrot inside and were discreetly refilled between each trial with the lid put back on while the horse was walking in the opposite direction. To point at the bucket, the informant moved one step from the centre of the two buckets towards one of them and pointed at it with their back slightly bent and hand about 30 cm above the bucket, staring at the bucket as they did so (Fig. 1d and picture). The idea was to mimic the natural way humans would inform an animal about the location of an object. The informant pointed with their right arm when pointing at the bucket on their right and used the left arm to point at the bucket on their left. The horse was then released and free to choose one of the buckets. In a successful trial, the horse chose the bucket pointed at by the informant by touching the lid with its muzzle or by smelling it with its muzzle within 10 cm, and the informant then quickly opened the lid and the horse got to eat the carrot inside. Thereafter, an assistant led the horse back to the starting point for the next trial. In a failed trial, the horse chose the bucket not pointed out by the informant. In that case, the informant caught the horse by the halter and did not open the lid of the bucket. The assistant led the horse back to the starting point without a reward. The informant then performed a motivational trial before continuing with the next pointing trial to prevent any frustration among the horses. The motivational trial consisted of one repeat of training phase *c* (Fig. 1c). In total, ten pointing trials were performed—not including the potential motivation trials which were performed after each failed trial. All trials were performed one after another and the horses had one minute to pick a side. The side of the pointing was pseudo-randomised, so each side was pointed to five times, and the same bucket was never pointed at more than twice in a row. The informant was randomly selected and was either the person familiar to the horse or one of the four unfamiliar experimenters. The identity of the unfamiliar informant was pseudo-randomised depending on the availability of the experimenters (number of horses tested by each unfamiliar informant: OL = 11, AV = 6, VR = 6, EA = 6). In total, 29 horses were tested by an unfamiliar informant and 28 horses were tested by a familiar person (see Table 2). Of the 57 horses which performed the test, 56 completed all 10 trials, with one mare completing only 9 because she got distracted during the last trial and did not focus on the task after becoming distracted.

All tests were video recorded for later analysis of the success using the Behavioural Observation Research Interactive Software (BORIS) (Friard and Gamba 2016). Two observers coded the videos to test the inter-observer reliability but considering the horses' choices were unambiguous, observers had 100% inter-observer reliability.

### Sanitary protocol

To prevent the transmission of horse pathogens, subjects were handled with their own halter, the experimenters’ shoes were washed and sprayed with ©Virkon disinfectant, the buckets were washed, and the experimenters’ clothing was washed in a washing machine with neutral scent detergent in between each stable. The barrier gestures regarding the Covid 19 virus were applied according to the recommendations of the Finnish government at the time of the experiments.

### Statistical analysis

Statistical analyses were performed using the statistical software R, version 3.6.3 (R Core Team 2022) and figures were created using the *ggplot2* package (Wickham 2016).

We checked for potential collinearity between our variables of interest (relationship length, social environment, physical environment, age and sex) using Spearman and *χ*^2^ correlation tests. There was a significant moderate correlation between the relationship length and the age of the horse (Spearman, *r*_s_ = 0.49, *P* < 0.0001, *N* = 57), but this was below a collinearity level of *r*_s_ < 0.7 generally considered as problematic for modelling both parameters simultaneously, and both were therefore kept in the same model (Zuur et al. 2010). There is also a strong association between the social and physical environments (*χ*^2^ = 45.18, *df* = 4, *p* < 0.0001, *N* = 57). This association is difficult to avoid since horses living in stalls or small paddocks (group: Paddock) are kept individually most of the time (group: Alone) (*n* = 13) and horses living in groups (group: Group) are often kept in a bigger field (group: Field) for space reasons (*n* = 23). However, a contingence table (Table 3) shows that a total of 26 horses were housed in different configurations (e.g. groups in paddocks/fields, dyads in fields or paddocks, horses alone in paddocks/fields), making it difficult to group the two variables into one without losing half of the data. Therefore, we decided to explore these two factors independently to allow us to investigate them more precisely by implementing the analyses with the effects of the dyads and the alternation between paddocks and fields (see Table 3). The fact that these two variables are highly correlated will be considered when discussing the results.

**Table 3:**
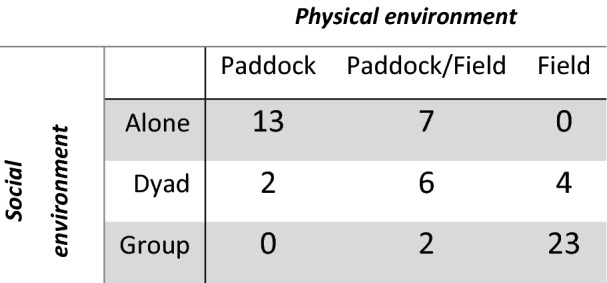
Contingency table presenting the dependence between the social and physical environments of the horses

We investigated the horses’ success in the pointing task with their success in each trial as the response variable (success vs failure). We included the informant familiarity (2-levels factor: owner vs unknown experimenter), relationship length (continuous variable ranging from 6 months to 14 years), social environment (3-levels factors: alone, in dyads or in groups of 3 or more), physical environment (3-levels factors; paddock, paddock and field, and field only), age (continuous variable ranging from 2 to 26 years old) and sex (2-levels factor: females vs males) as independent variables, as well as all interactions between the informant familiarity and other variables. We used Generalised Linear Mixed Models (GLMM) from the package ‘glmmTMB’ (Brooks et al. 2017) using a binomial distribution with a logit link function. We added the trial number as a random factor to control for any learning effects over the 10 trials. As each horse went through 10 trials, the horse identity was also added as a random factor to account for the repeatability. Since several horses were housed at the same stable and some owners participated with several horses, the horse identity was nested in the owner identity nested in the stable identity. Because of the association between the physical and social environment we designed two separate models:

- *Initial model1* Trial Success ~ Informant Familiarity * relationship length + social environment + age + sex + (1|Stable_ID/Owner_ID/horse ID) + (1|Trial Number)
- *Initial model2* Trial Success ~ Informant Familiarity * relationship length + physical environment + age + sex + (1|Stable_ID/Owner_ID/horse ID) + (1|Trial Number)
- We tested for a potential learning effect through the 10 trials with a model that included the Trial Success as the response variable (yes/ no) depending on the trial number in interaction with the informant familiarity to test the prediction that horses may learn faster when informed by someone familiar. The Stable Identity, owner identity and the Horse identity were again included as nested random factors to control for repeats within each study participant.
- *Initial learning model* Trial Success ~ Trial Number * Informant Familiarity + (1|Stable_ID/Owner_ID/horse ID)

Significance was evaluated at 0.95 (*P* < 0.05). The result figures are based on the estimated marginal means of the response variables and their associated credible intervals extracted from the models using the function *emmeans()* from the ‘*emmeans*’ package (Lenth 2021).

## Results

### Overall success

Overall, the horses’ success at following the informant cue over the 10 trials ranged from 2 to 10 successes (mean ± SD: 7 ± 2).

### Informant’s familiarity and relationship length

Horses did not significantly differ in their success at following the human cue when the informant was familiar (72%) or unfamiliar to them (65%) (M1: estimate ± SE = 0.28 ± 0.45, *z* = 0.62, *p* = 0.537). The relationship length was also not significantly associated with the success rate of the horses (M1: estimate ± SE = − 0.04 ± 0.08, *z* = − 0.57, *p* = 0.548) and it also did not interact with the informant familiarity (M1: estimate ± SE = − 0.13 ± 0.09, *z* = − 1.55, *p* = 0.121), meaning that horses did not perform better with the familiar person giving them cues than with an unfamiliar informant, regardless of how long they had known each other (at least 6 months in the sample).

### Social environment and housing

**Fig. 2:**
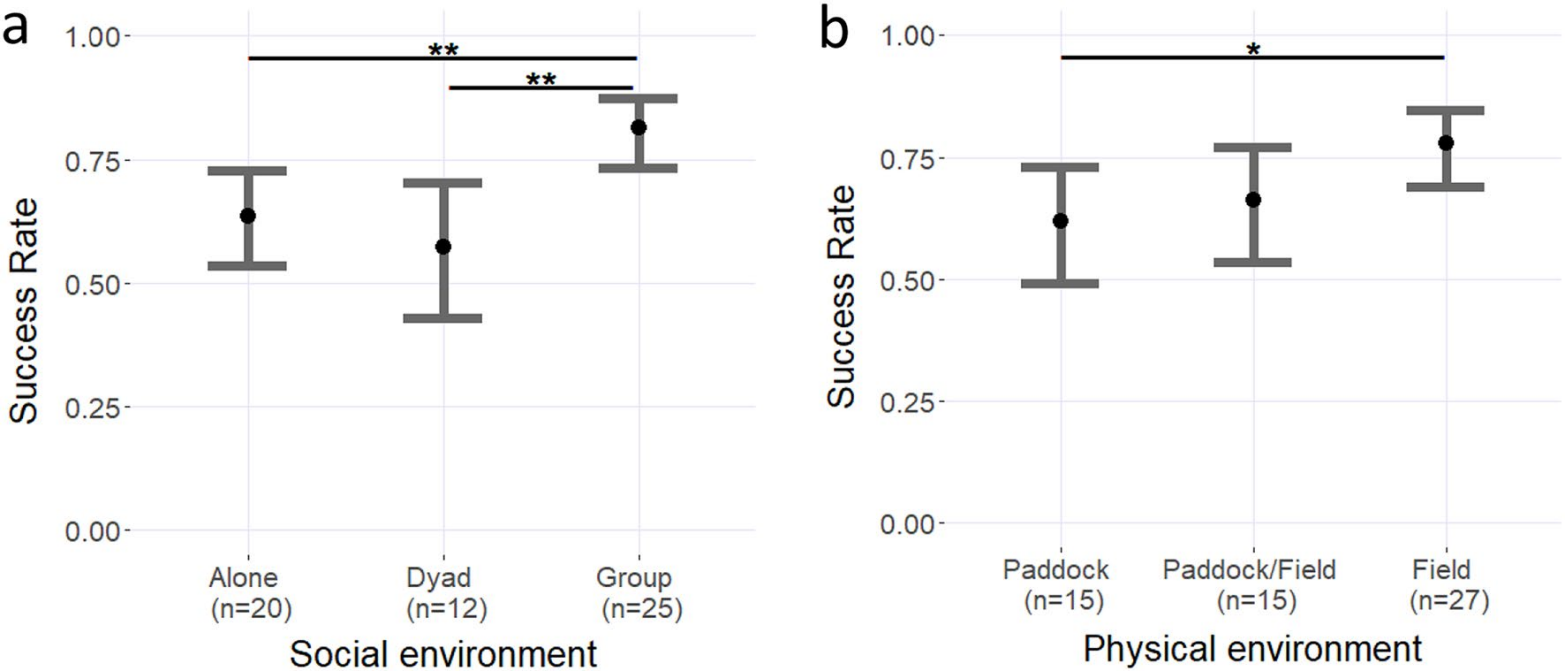
**a** Effect of the social environment of the horses on their success in the pointing task. **b** Effect of the horses’ physical environments on their success in the pointing task. The error bars represent the 95% confidence intervals. ‘*’ indicates significant differences with a *p*-value < 0.05. ‘**’ Indicates significant differences with a *p*-value < 0.01. The means and their associated 95% confidence intervals were extracted from the models M1 and M2 using the function *emmeans* from the “emmeans” package

The social environment of the horses was significantly associated with their success at following human cues, regardless of the informant’s familiarity. Horses living in groups had a significantly higher success rate (81%) compared to horses kept alone (64%) (M1:Estimate ± SE = − 0.91 ± 0.08, *z* = – 2.86, *p* = 0.004) and horses kept in dyads (57%) (M1: Estimate ± SE = – 1.18 ± 0.37, *z* = – 3.17, *p* = 0.002). There was no significant difference between horses living alone or in dyads (M1: Estimate ± SE = – 0.27 ± 0.35, *z* = – 0.77, *p* = 0.444) (Fig. 2a).

The physical environment of horses was also significantly associated with their success rate, regardless of the informant’s familiarity. Horses living most of their time in stalls and paddocks had significantly lower success rate (62%) compared to horses spending all year in bigger fields (79%) (M2: Estimate ± SE = – 0.77 ± 0.35, *z* = – 2.24, *p* = 0.025). There were no significant differences between horses alternating between paddocks and fields (64%) and horses spending all year in bigger fields (M2: Estimate ± SE = – 0.58 ± 0.35, *z* = – 1.64, *p* = 0.106). There were no significant differences between the horses living in paddocks most of the year and horses alternating between paddocks and fields (M2: Estimate ± SE = 0.19 ± 0.38, *z* = – 0.51, *p* = 0.607) (Fig. 2b).

### Age and sex

**Fig. 3:**
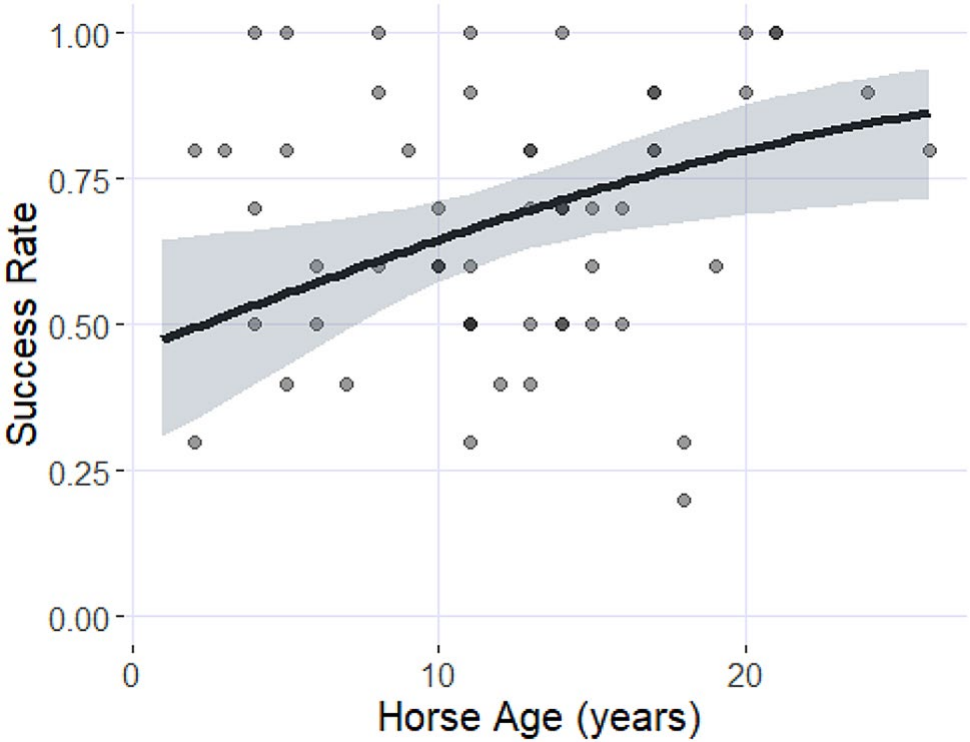
Effect of the horses age on their success in the pointing task. The shaded area represents the 95% confidence intervals. The graph is extracted from the post-hoc Tukey tests performed on the selected model 1

The horse success in the pointing task significantly increased with the age of the horses (M1: estimate ± SE = 0.08 ± 0.03, *z* = 2.53, *p* = 0.011). The post-hoc analyses revealed that the success rate increased from 47% success rate at age = 1 year to 86% at age = 26 years (Fig. 3). There was no significant effect of the sex on the horse success in the pointing task (M1: estimate ± SE = – 0.16 ± 0.26, *z* = – 0.62, *p* = 0.532).

### Learning performance

The success of the horses did not improve along the 10 pointing trials (M3: estimate ± SE = 0.06 ± 0.05, *z* = 1.21, *p* = 0.227). The learning performance across the 10 trials did not significantly differ according to the informant familiarity (M3 interaction: estimate ± SE = – 0.04 ± 0.07, *z* = – 0.65, *p* = 0.519).

## Discussion

In this study, we explored factors that could be associated with the performance of horses in a socio-cognitive task involving communication with humans. First, we hypothesised that horses would perform better with familiar informants because they are already used to working with them, and that those with longer relationships with the familiar person would perform better than those with shorter relationships. Interestingly, the informant identity and the relationship length with the main caretaker were not associated with the horses’ performance, but the horse performance did improve as the horses were older. We also hypothesised that the horses’ living conditions affecting their social environment and freedom of movement may interfere with the horses’ socio-cognitive abilities towards humans. Our results support part of our hypotheses and suggest that an appropriate living environment may be involved in an improved used of the human-given cues. Therefore, such aspects should be considered in studies evaluating animal behaviour.

### Familiarity and relationship length with the informant

Recreational animals such as horses in riding schools but also tourism animals (e.g. camels, Asian elephants or sledge reindeer and dogs) can be confronted with numerous daily interactions with strangers, and interacting with unfamiliar humans may even form a key part of their life. They must cope with the requests of many different persons with different levels of experience in handling those animals and different ways of expressing their intentions. However, privately owned animals used for leisure activities or competition can also develop a relationship with one or a limited number of persons, but they also often must cope with owner changes up to several times in their life, meaning that they have to repeatedly develop a relationship with a new human, which can take time. Investigating how familiarity and relationship length with the handler affect a horse’s ability to correctly understand and interact with the handler is therefore essential, as communication is a crucial part of the development of their relationship.

Interestingly, in our experiment, neither the familiarity of the informant or the relationship length between the horse and the familiar person statistically affected the horse performance during the task. This is congruent with the findings of Krueger et al. (2011) who found in a three-choice task that even if horses focussed their attention more on familiar than unfamiliar informants, the horses did not perform better with familiar informants. Similarly, in 2017 Scandurra et al. showed that for dogs, their responses to gestural stimuli are independent from the informant familiarity.

In experiments based on the same population of horses as in this study, Liehrmann et al. (2022) found that older horses agreed more often to walk on a novel surface when led by someone familiar than someone unfamiliar, and horses with longer relationship with their owner performed less reluctant behaviours towards the novel objects than horses having shorter relationship with their caretaker. Similarly, working Asian elephants agreed more often to step on a novel surface with and handler they knew for more than a year (Liehrmann et al. 2021). In our study, the experiment involved free participation from the horses and included food reward so the informant was probably quickly associated with something positive. In Liehrmann et al. (2021, 2022) Asian elephants and horses were asked to interact with a novel object which is generally used as a test to assess the fearfulness and the neophobia of individuals (Dalmau et al. 2009; Lansade et al. 2008; Leiner and Fendt 2011; Sneddon et al. 2003), therefore this is a more stressful situation. Our hypothesis is that the context may play a role when investigating the effect of human familiarity in human–animal interactions. In a more stressful environment animals may rely more on a familiar human than on a stranger as they act as a secure base to them, while in a positive context where animals already feel safe, then the identity of the interacting human may matter less. This theory is supported by the findings of Kerepesi et al. (2015). In their study on dogs they observed that dogs did not show difference in responding to different partners in obedience tasks but relied more on their owner than a familiar or unfamiliar person in situations provoking anxiety. They conclude that the discrimination between the owner and a less familiar person is context specific. Further studies should investigate the role of the familiarity with the caretaker in various contexts. More generally, the experimenter’s familiarity should be given more consideration in studies experimentally investigating animals’ socio-cognitive skills and human–animal interactions.

### Social and physical environment

Domestic horses do not benefit from the same freedom of movement or have the same choice in the composition of their social groups as feral horses do. Some horses can be kept in individual stalls with a limited amount of time outside per day, and other horses live all year-round in groups in pastures of several hectares. Hockenhull and Creighton (2014) report that the main source of behavioural problems and aggressiveness towards humans in leisure horses is their husbandry. Investigating how housing variation affects horses’ behaviour and interactions with humans have therefore become a topic of concern for the stakeholders related to equine behaviour and welfare, such as the national and international equestrian federations. In our study, both the social and physical environment were strongly associated with the horses’ success at following the human-given cues. Horses living in groups had a higher, 82% success rate in the pointing task compared to horses living in dyads (57%) or living alone (63%). Similarly, horses living in stalls or small paddocks for most of the year had only about a 62% success rate in the pointing task, and horses alternating paddock and bigger summer fields reached 64% success rate while horses living in a bigger field all year long reached a 79% success rate. Of course, both housing factors are strongly associated, and it is difficult to conclude whether social deprivation or the lack of space and enrichment has the biggest impact on the results. Nevertheless, both factors relate to the quality of the living environment of the horses and can reflect on their potential welfare state. These results therefore make an important contribution to the existing literature associating poorer welfare with lower cognitive performance in horses (Hausberger et al. 2019).

Hockenhull and Creighton (2014) highlighted that for horses, being able to see other horses from the stable but not being able to have full-body contact with them may generate even more frustration than when there is no visual contact at all. Horses housed individually may have suffered from social deprivation in comparison to horses living in groups composed of more than two individuals, even though they had visual contact and sometimes could interact slightly with conspecifics through the fences. Lee et al. (2011) showed that when horses were housed in individual stalls and they were given the choice to go back to their stall or to the paddock every 15 min, the horses chose to stay longer outside when there were conspecifics in the paddock. They would also stay much longer outside with conspecifics after a 48 h deprivation of stall release. In our study, horses living in dyads also had lower success rates. Domestic horses living in larger groups may benefit from stronger cognitive stimulation. Indeed, having the choice of interacting with various conspecifics promotes complex social situations from which horses can learn and improve their socio-cognitive skills, potentially explaining the better success in the pointing task of horses who lived in larger groups. Temporary social changes have been shown to have a direct impact on animals’ brain structure even outside of the critical developmental stages. Sallet et al. (2011) observed that after 4 months, rhesus macaques (*Macaca mulatta*) housed in larger groups had an increased amount of brain grey matter compared to individuals housed in smaller groups. In addition, several studies comparing rats reared in isolation versus in groups found positive effects of sociality on cognitive skills (Ashton et al. 2018a). In another study Ashton et al. (2018b) revealed that in free-ranging Australian magpies *(Gymnorhina tibicen*), the individual’s performance in a set of four cognitive tasks increased with the size of their social group. These studies suggest that for social species, living in larger groups may contribute to their cognitive development. In 2014 Lansade et al. showed that an enriched environment (access to pasture with conspecifics) could promote better performance in learning tasks, based on understanding of informing cues. Our results support the idea that housing horses in groups and in pasture could contribute to their cognitive development and improve their socio-cognitive abilities towards humans.

### Horse age

Our study did not demonstrate the potential benefits of a longer relationship length with the main caretaker on the horses’ success at following the human-given cues. The horse success rate at the task did not improve with the relationship length with the familiar informant but did increase with the age of the horses. These results support the idea that through their life leisure horses are experiencing more and more interactions with a various number of different humans from which they may be getting better at generalising human communicative cues. This acquired knowledge could explain that, with age, horses get better at following human-given indications regardless of who the human informant is and for how long they have known each other. Another explanation could be that older horses may be more attentive and get less distracted than younger horses. In Ringhofer et al. (2021), horses with higher sustained attention levels could evaluate the credibility of a human information and followed the pointing of an informant who knew where food was hidden. We did not assess the attention level of the horses in our study and did not find studies supporting that young horses have lower attentional state than older horses. Moreover, the use of food reward is likely to enhance the attention towards the experimenter in 1 to 2-year-old horses (Rochais et al. 2014). Our results are contradictory with previous findings. In dogs (*n* = 16) (Agnetta, et al. 2000) which did not observe improvement with the age of dogs (4 months–4 years) when following ostensive human-given cues. In goats (*n* = 23) Kaminski et al. (2005) found that young goats (4–6 months), were as skilful at using ostensive pointing cues as were the adults (2–11 years). However, in both studies the sample sizes were relatively small, and the age range did not cover the old individuals. Even though we did not use ostensive communicative cues, our data suggests that the best performers in using human-given cues are the older horses (> 19 years old, *N* = 7) which all scored with at least 80% of the success rate. There is a need for studies covering a wider age range when investigating the human–animal interactions as the experience of older individuals may be underestimated.

### Learning effect

There was no learning effect as our results do not show any improvement of success over the 10 trials in the pointing task. This result may seem surprising as we would expect the horses to learn from their mistakes and not to fail anymore at the end of the pointing task. Because the informants were using a combination of pointing, gazing and were closer to the target, the horses could rely on multiple cues to understand the intention of the informant, making the task easier than in other studies focussing on one type of cue (Krueger et al 2011; Proops et al. 2010, 2013). In Cunningham and Ramos (2014) dogs had an easier time to follow cues where gesture was combined with a congruent head and eye movement compared to either gesture or eye gaze alone.

## Conclusion

Overall horses’ success rate at following the informant given cues improved with the horse age and was not affected by their relationship length with the familiar informant or the informant familiarity. This indicates that the horses may learn overtime how to read human-given cues and apply it to familiar and unfamiliar humans. Second, this study shows that the living conditions of the horses had an impact on their ability to follow human indication. The housing and social environment of horses is a challenge for the stakeholders related to equine behaviour and welfare. Our results support the idea that offering an appropriate environment to horses, by providing access to pasture and the ability to freely interact with conspecifics could contribute to the development of socio-cognitive abilities of individuals towards humans. Overall, more research is needed to assess the mechanism underlying the effects of extrinsic factors on the cognitive abilities of long-lived social species.

## Data Availability

The datasets generated during and analysed during the current study are available in the Mendeley Data repository: Liehrmann (2023), “Familiarity, environment and the use of human-given cues in horses”, Mendeley Data, V1, doi: 10.17632/6hg6c7kt3z.1.
